# Warming and Ocean Acidification Effects on Phytoplankton—From Species Shifts to Size Shifts within Species in a Mesocosm Experiment

**DOI:** 10.1371/journal.pone.0125239

**Published:** 2015-05-20

**Authors:** Ulrich Sommer, Carolin Paul, Maria Moustaka-Gouni

**Affiliations:** 1 Marine Ecology, GEOMAR Helmholtz Centre of Ocean Research Kiel, Kiel, Germany; 2 School of Biology, Aristotle University of Thessaloniki, Thessaloniki, Greece; Fudan University, CHINA

## Abstract

While the isolated responses of marine phytoplankton to climate warming and to ocean acidification have been studied intensively, studies on the combined effect of both aspects of Global Change are still scarce. Therefore, we performed a mesocosm experiment with a factorial combination of temperature (9 and 15°C) and pCO_2_ (means: 439 ppm and 1040 ppm) with a natural autumn plankton community from the western Baltic Sea. Temporal trajectories of total biomass and of the biomass of the most important higher taxa followed similar patterns in all treatments. When averaging over the entire time course, phytoplankton biomass decreased with warming and increased with CO_2_ under warm conditions. The contribution of the two dominant higher phytoplankton taxa (diatoms and cryptophytes) and of the 4 most important species (3 diatoms, 1 cryptophyte) did not respond to the experimental treatments. Taxonomic composition of phytoplankton showed only responses at the level of subdominant and rare species. Phytoplankton cell sizes increased with CO_2_ addition and decreased with warming. Both effects were stronger for larger species. Warming effects were stronger than CO_2_ effects and tended to counteract each other. Phytoplankton communities without calcifying species and exposed to short-term variation of CO_2_ seem to be rather resistant to ocean acidification.

## Introduction

The well known increase of atmospheric CO_2_ does not only lead to climate warming because of the greenhouse effect but also to “ocean acidification”, i.e. an increase of dissolved CO_2_, a decrease of water pH and a decrease in the saturation state of calcium carbonates in the ocean. Current predictions for atmospheric CO_2_ assume an increase from approximately 390 ppm to 700 ppm by the end of 21^st^ Century (RCP8.5 scenario of the IPCC report 2013 [1]). The same scenario also predicts temperature increases of up to 6°C. The predicted increase in CO_2_ will lead to a further pH decrease by 0.3–0.4 units [2], while until today, ocean pH has declined by 0.1 units from pre-industrial level and carbonate ion concentration have decreased by 30% [3]. The shift in carbonate chemistry is primarily a stressor for organisms with skeletal calcium carbonate structures [4], i.e. among phytoplankton coccolithophores should be affected most strongly [5,6,7].

For non-calcifying phytoplankton, however, CO_2_ might be a limiting resource because of the low pCO_2_ under ocean pH and the low affinity of the enzyme RubisCO for dissolved CO_2_ [8]. While phytoplankton have evolved carbon-concentrating mechanisms (CCM) to overcome this problem [9,10,11], increased CO_2_ concentration might still be beneficial because they could help to save metabolic costs for CCMs. Indeed, Riebesell [12] found enhanced carbon assimilation of phytoplankton in mesocosms receiving enhanced CO_2_ concentrations. There is some evidence, that CCM efficiency and regulation differs between taxa [13,14] which should lead to the prediction, that phytoplankton composition is sensitive to CO_2_-enrichment if CO_2_ is a limiting factor. In a review article, [15] it was proposed that coccolithophores should be negatively affected by increasing CO_2_, for diatoms it should be neutral or slightly beneficial, and for N_2_-fixing cyanobacteria strongly beneficial. Tortell [16] found a shift from small pennate diatoms (*Pseudo-nitzschia subcurvata* G.A. Fryxell) to large centric ones (*Chaetoceros* spp.) under enhanced CO_2_. Similarly, Eggers [17] found that CO_2_ enrichment could favour large diatoms (*Chaetoceros sp*., *Thalassiosira constricta* Gaardner) depending on initial species composition. In contrast, only subtle changes in species composition were reported in [18]. A recent review of ocean acidification effects on marine pelagic microbes [19] emphasizes an insufficient state of knowledge, conflicting results between different studies and a tendency of effects to be rather subtle. The latter is inter alia explained by the fact, that present day seawater pCO_2_ and pH undergo short-term and seasonal fluctuations which often exceed the expected increase of the atmospheric input.

The previously mentioned review [19] also emphasizes the need to understand the joint effects of ocean acidification with other aspect of Global Change, in particular climate warming. We have accepted this challenge and designed one of the first large volume (1400 L) mesocosm experiments with a crossed factorial design of temperature and CO_2_ enrichment (but see [20] for a smaller scale experiment). The experiment was performed in area where phytoplankton contains no coccolithophores and where plankton is already now subject to strong short term and seasonal variations in pCO_2_ (summer and autumn maxima ca. 2300 ppm, summer and autumn means ca. 700 ppm [21]). Therefore, we expected no direct detrimental effect of CO_2_ on phytoplankton, while indirect food-web effects could not be excluded a priori. The predictions for the effects of the temperature treatment were derived from previous experiments performed at the same site, but in a different season (summarized in [22]). We extended the analysis from species shifts to size shifts within species, because several experiments have indicated a shrinkage of cell size with warming [23,24] and nutrient stress [25]. We chose autumn as the season of our experiment, because it is usually the period of maximal phytoplankton diversity in our region, in particular because of the mixture between diatoms and flagellates of various phylogenetic origin. Our working hypotheses are:

1. Warming will lead to decreased phytoplankton biomass, in agreement with previous experimental results and generally explained by a stronger effect of warming on loss terms than on photosynthesis, as summarized in [22].
2. Addition of CO_2_ will lead to increased phytoplankton biomass, because acidification sensitive coccolithophores [5,6] are lacking in the Baltic Sea and CO_2_ might be a limiting resource [6,12].
3. Warming will lead to shifts in taxonomic composition of phytoplankton as observed in previous experiments [summarized in 22].
4. Addition of CO_2_ will lead to taxonomic shifts in phytoplankton because of taxonomic differences in CCM [13,14] and of previous experimental results [15,17, but see 18].
5. Warming will lead to smaller cell sizes of phytoplankton and heterotrophic nanoflagellates in agreement with previous experimental findings [23,24]
6. Addition of CO_2_ will lead to bigger cell sizes of phytoplankton, but not of heterotrophic protists. We expect this response because of the analogy to the limiting nutrient effect on phytoplankton cell size [25] while CO_2_ is not a source of nutrient for heterotrophic nanoflagellates.

## Material and Methods

The field samples taken for our study did not involve protected species and were not taken from a protected area. No permissions were required.

### Experimental design

The experiment consisted of 12 mesocosms in temperature controlled rooms of GEOMAR-Kiel. We employed a fully factorial combination of two temperature levels (9 and 15°C, in situ temperature was 12°C) and two CO_2_ levels (target values 560 ppm and 1400 ppm CO_2_). Target values of CO_2_ were chosen to represent present days annual minima of the Kiel Bight and the mean value expected for 2100 assuming the more pessimistic IPCC-scenarios for 2100 [1]. Each treatment was replicated three times. In the following text, the experimental treatments will be characterized by a two-letter code (CL: cold, low CO_2_, CH: cold, high CO_2_, WL: warm, low CO_2_, WH: warm, high CO_2_). The mesocosms had a volume of 1400 L. In order to assure a homogenous distribution of plankton and to minimize sedimentation, mesocosms were gently and continuously stirred by a propeller. A series of previous experiments with the same mesocosms [22,26,27] has shown that this treatment did not harm our experimental organisms.

The mesocosms were illuminated by computer controlled light units (GHL Groß Hard- und Softwarelösungen, Lampunit HL3700 and ProfiluxII) consisting of 5 HIBay-LED spotlights (purpose build item of Econlux, each 100 W) above each mesocosm. The lamps cover the entire PAR spectrum and have emission peaks close to the blue absorption peak of chlorophyll a and to the absorption peak of xanthophylls. Daily irradiance patterns were set to follow the pattern for a cloudless 21 October at Kiel (calculated according [28]) reduced to 50% to account for moderate under water light attenuation. The day-night cycle was 11h50 min: 12h10 min and noon irradiance in the middle of the water column was 252.3 μmol quanta m^-2^ s^-1^ PAR at the start of the experiment. The impact of temporal changes in underwater light attenuation due to growth and decline of phytoplankton biomass was minor, because the mesocosms were only 1 m deep.

The mesocosms were filled with unfiltered natural seawater (salinity: 19.7, in situ SST 12°C) from Kiel Bight, Western Baltic Sea (54°19’45.99”N, 10°08’58.19”E) near the dock of the GEOMAR West Shore Building on 19 October 2012. The water contained the natural autumn community of phytoplankton, bacteria, and protozoa. Mesozooplankton were added from net catches with a target density of 20 individuals L^-1^, mimicking seasonal maximum levels. CO_2_ was manipulated by a flow-through of air-CO_2_-mixtures with 560 and 2400 ppm CO_2_ through the head-space between the water level and the cover of the mesocosms at a rate of 30–60 L h^-1^. Because of incomplete equilibration between the headspace and the water body and because of photosynthetic CO_2_-consumption mean values of pCO_2_ in the water were 439 ppm (sd = 187) and for high CO_2_ 1040 ppm (sd = 210) with peak values of 686 ppm and 1400 ppm. In the high CO_2_-treatments biological CO_2_-drawdown was counterbalanced by adding the required amount of CO_2_-saturated filtered mesocosm water at days 7, 12, and 19. The low CO_2_-mesocosms received the same amount of filtered water, but without CO_2_ addition. Full divergence of temperature and CO_2_ between the treatments was reached on 22 October, henceforth called day 0, while the filling day will be called day -3. The experiment was terminated on 12 November.

### Samples

Water temperature, salinity and pH were monitored daily. DIC and total alkalinity were measured and pCO_2_ was calculated 3 times per week. Details of the methodology and of the temporal change of pH and the carbonate system are available from the data archive and will be published by C. Paul et al. (submitted).

Sampling for phytoplankton took place on three times per week on Mondays, Wednesdays, and Fridays. Samples were taken with a bucket after mixing the mesocosms. Samples for phytoplankton >5 μm were fixed with Lugol’s iodine for subsequent identification, counting (>100 individuals for common taxa), and sizing by inverted microscopy [29]. For phytoplankton <5 μm, 20 ml water samples pre-filtered with a 64 μm mesh were fixed with formaldehyde, incubated, filtered onto black Nuclepore filters (0.8 μm pore size) and stained with DAPI [30] within a few hours of sampling and stored at -80°C until counting and sizing. Filters were examined using fluorescence microscopy under blue and green excitation at 1000X. Dimensions were measured on freeze-frame micrographs of individuals using the Nikon DS-L1 digital camera tools (Nikon, Tokyo, Japan). An unfixed aliquot was immediately analysed by a flow cytometer (FACScalibur, Becton Dickinson) and distinguished on the basis of pigment fluorescence (chlorophyll a, phycoerythrine) and size (equivalent spherical diameter). Flow-cytometric categories were matched to taxa identified by fluorescence microscopy on the basis of size and correlations between abundances. In addition, small phytoplankton not identifiable by microscopy were identified by means of 18S rRNA gene tag pyrosequencing according to [31].

Sizing of phytoplankton was performed at three levels of effort. The flow cytometer delivered size data for each sample individually. Common phytoplankton taxa >5 μm were sized individually for each mesocosm at the time of their abundance peak. Twenty random cells were measured per sample. Taxa which were too rare for this kind of measurement were measured in a composed sample from all mesocosms taken at 2 November (day 12) and, therefore, not used for the analysis of the size response of individual species. Their size data were only used for calculating biomass and together contributed <5% to total biomass. Appropriate geometric standard figures were used to calculate cell volumes from linear measurements [32]. We also measured the sizes of three sufficiently abundant heterotrophic nanoflagellates in order to see, whether the heterotrophic nanoflagellates would respond differently to the experimental treatments than phytoplankton.

### Statistical analysis

The responses to temperature and CO_2_ were analyzed by two-factor ANOVA. In each ANOVA, each mesocosms was represented by a single value (usually the mean over time) in order to avoid pseudoreplication. Biovolume (B) and individual cell volume (V) data were log-transformed and relative biomass data of taxa (p_i_ = B_i_/B_tot_) were arcsine-square root transformed. We also calculated effect sizes for temperature (E_T_) and CO_2_ (E_c_) on cell volumes by calculating the log-ratios between the mean values for the different treatment levels, i.e. the mean cell volumes of a given species for the mesocosms receiving the same temperarure (E_T_) or the same CO2-treatement (E_C_):

$$E_{T}=\log^{e}(\frac{meanV_{15}}{meanV_{9}})$$

$$E_{C}=\log^{e}(\frac{meanV_{high}}{meanV_{low}})$$

## Results

### Phytoplankton biovolume

**Fig 1:**
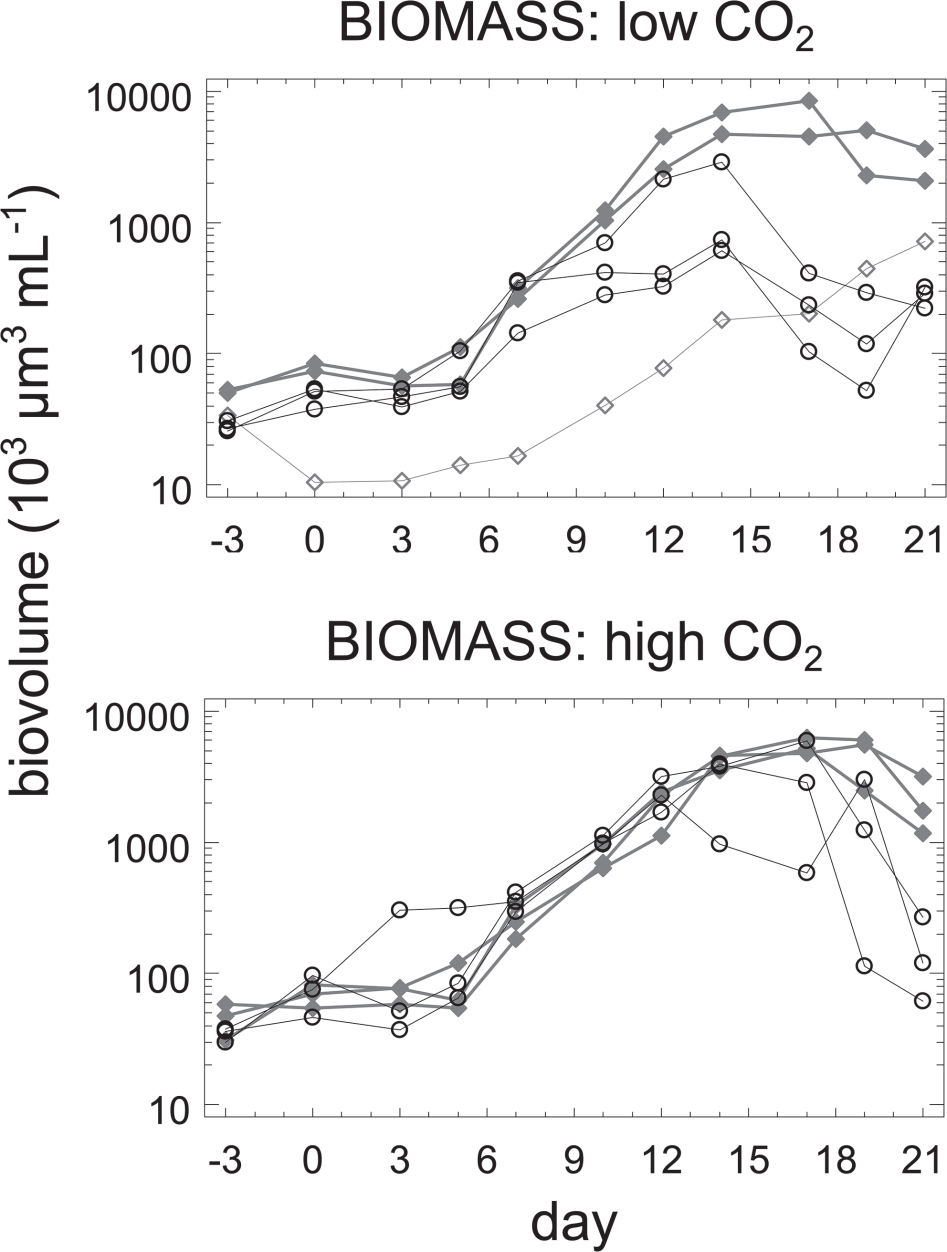
Time course of phytoplankton biovolumes. Open circles: warm mesocosms; grey and filled diamonds: cold mesocosms; open diamonds: mesocosms 9.

**Fig 2:**
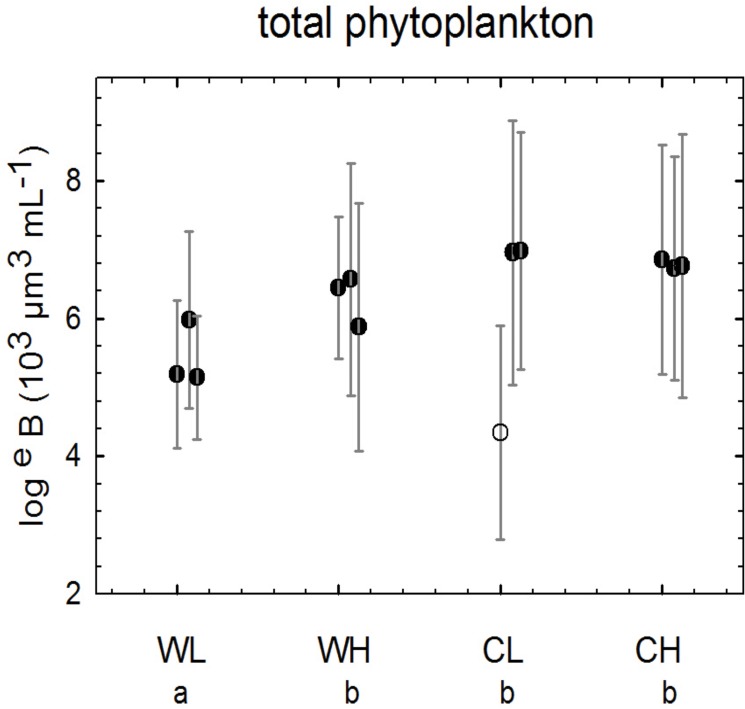
Means of total phytoplankton biomass. Means over entire experiment calculated after log^e^-transformation and standard deviations (vertical lines). Mesocosm is 9 shown by an open symbol. Treatment codes: WL: warm, low CO_2_, WH: warm, high CO_2_, CL: cold, low CO_2_, CH: cold, high CO_2_; codes for homogenous groups according to Tukey’s HSD: a, b.

In most mesocosms, appreciable growth of biomass began in the interval from day 3 on, except for mesocosm 9, one of the CL mesocosms (Fig 1). In this mesocosm, there was a 72 hrs failure of the light supply at the beginning of the experiment which initiated a completely aberrant trajectory of plankton dynamics. This mesocosm was excluded from further analysis. At low CO_2_, warm and cold mesocosms began to diverge during the period from day 10 to 14when growth was maintained in the cold mesocosms but began to slow down in the warm ones. From day 14 on, biomass began to decline in the warm mesocosm to levels almost one order lower than maximal ones for the warm treatments. In the cold mesocosms higher maximal levels were reached, decline started on day 16 and was less pronounced. In the high CO_2_ mesocosm divergence between cold and warm ones was observed towards the end of the experiment (days 19 to 21) and the differences between peak values of warm and cold mesocosms were less pronounced than in the low CO_2_ treatments. A two-factor ANOVA based on mean values over the entire experiment (Fig 2) showed a significant effect of temperature (p = 0.0014), no significant effect of CO_2_, and a significant interaction effect (p = 0.0027). A multiple range test showed two homogenous groups, one consisting of CL, CH, and WH treatments and one of the WL treatments with significantly lower biovolumes.

### Species composition

**Fig 3:**
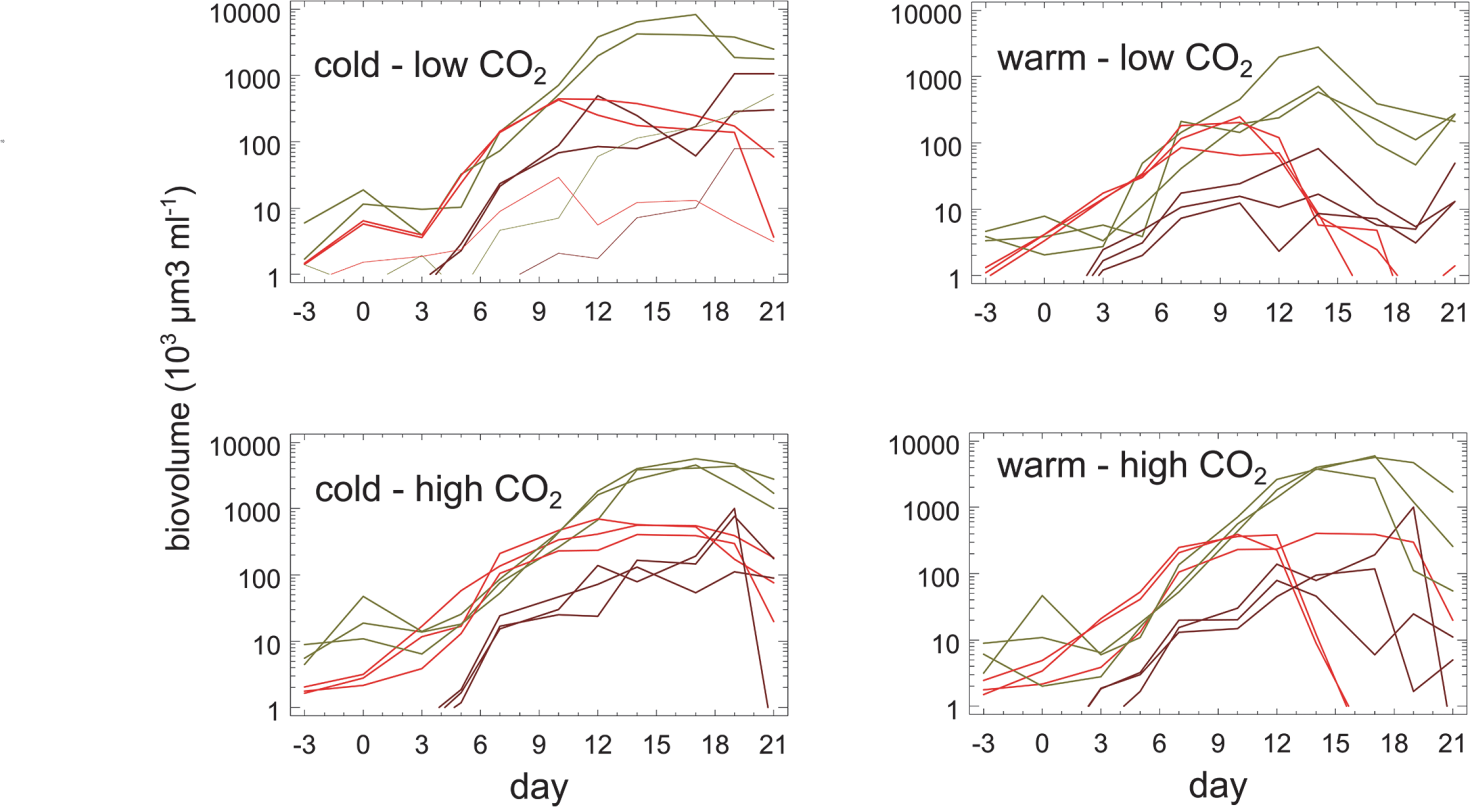
Time course of the biovolumes of dominant higher taxa. Diatoms (olive), cryptophytes (red), and prymnesiophytes (brown), the trajectories from the aberrant mesocosm 9 are shown by dotted lines.

The succession patterns of higher phytoplankton taxa did not differ strongly between the different treatments (shown for diatoms, cryptophytes, and prymnesiophytes in Fig 3). Initially, large dinoflagellates (*Ceratium* spp.) together with large diatoms (*Ditylum brightwelli* Grunow, *Proboscia alata* Sundström, *Rhizosolenia setigera* Brightwell) dominated biomass. The large dinoflagellates stagnated until day 3 to 5 and then began to decline slowly, possibly because of the mixing of the mesocosms. The large diatoms grew slowly, but were quickly surpassed by the small celled *Skeletonema marinoi* Sarno & Zingone which together with extremely large *Coscinodiscus wailesii* Gran & Angst made up for most of the diatoms growth observed in the mesocosms. Until ca. day 9, cryptophyte growth (mainly *Teleaulax acuta* D.R.A. Hill) kept pace with the diatoms and in some cases even surpassed them, but thereafter cryptophytes either stagnated or declined. They were replaced by abundant prymnesiophytes (2 spp. of *Chrysochromulina*), but prymnesiophyte biomass never surpassed diatom biomass. At the level of higher taxa, the biomass trajectories in the aberrant mesocosm 9 looked almost like a delayed and miniaturized copy of the other mesocosms.

The relative biomass of the two dominant higher taxa, diatoms (grand mean 83% of total biomass) and cryptophytes (grand mean 10.5%) did not respond to warming and acidification. The relative biomass of prymnesiophytes (grand mean 4.7%) responded negatively to warming, while the relative biomass of dinophytes (1.5%), pico-chlorophytes (0.6%) and pico-cyanobacteria (0.14%) responded positively to warming. Dinophytes responded also negatively to acidification and showed a significant interaction effect between temperature and CO_2_. The other higher taxa did not show any significant response to CO_2_ (Table 1).

**Table 1:** Relative biomass of higher taxa and species.

	Grand mean pi	sign-temp	sign-CO_2_	F-temp	F-CO_2_	F-int
BACILLARIOPHYCEAE	0.826			0.68^ns^	0.97^ns^	3.22^ns^
*Skeletonema marinoi* Sarno & Zingone	0.627			0.87^ns^	1.32^ns^	0.52^ns^
*Coscinodiscus wailesii* Gran & Angst	0.061			2.15^ns^	0.97^ns^	0.65^ns^
*Rhizosolenia setigera* Brightwell	0.051			1.99	0.10	0.17
*Cerataulina pelagica* Hendey	0.019	-		**11.8** *	5.47^ns^	0.00^ns^
*Cylindrotheca closterium* Reimann & J. Lewis	0.016	+		**8.73** *	3.52^ns^	2.74^ns^
*Proboscia alata* Sundström	0.015	-	-	**9.53** *	**16.6** **	1.32^ns^
*Thalassiosira nordenskioeldii* Cleve	0.013	+		**7.15** *	0.00^ns^	1.25^ns^
*Thalassionema nitzschioides* Mereschkowski	0.012		-	0.07^ns^	**20.4** **	2.53^ns^
*Dactyliosolen fragilissimus* Hasle	0.004	-		**19.9** **	0.03^ns^	0.75^ns^
*Ditylum brightwellii* Grunow	0.003	+		**7.21** *	2.17^ns^	0.50^ns^
*Guinarida flaccida* H. Peragallo	0.002	-	-	**6.93** *	**11.5** *	0.00^ns^
*Chaetoceros* spp.	0.0006		-	0.41^ns^	**10.1** *	0.01^ns^
*Pseudontzschia* sp.	0.0002		-	1.21^ns^	**6.84** *	0.67^ns^
DINOPHYTA	0.015	+	-	**8.10** *	**7.40** *	**6.65** *
*Ceratium fusus* Dujardin	0.010		-	4.77^ns^	**8.14** **	**6.34** **
*Ceratium tripos* Nitzsch	0.004	+		**18.3** **	3.94^ns^	**6.93** *
*Ceratium lineatum* Cleve	0.0002		-	0.93^ns^	**8.74** *	1.34^ns^
*Prorocentrum micans* Ehrenberg	0.0001			5.15^ns^	3.69^ns^	0.71^ns^
CRYPTOPHYTA	0.105			0.20^ns^	0.04^ns^	5.11^ns^
*Teleaulax acuta* D.R.A.Hill	0.096			0.05^ns^	0.14^ns^	5.20^ns^
R5 = *Plagioselmis prolonga* Butcher	0.010			4.96^ns^	1.52^ns^	3.40^ns^
PRYMNESIOPHYTA						
R6 = *Chrysochromulina* spp.^1^	0.047	-		**11.5** *	2.48^ns^	0.41^ns^
CHLOROPHYTA	0.006	+		**11.9** *	1.49^ns^	**5.70** *
R1 = *Ostreococcus* sp.	0.004	+		**8.65** *	1.16^ns^	4.58^ns^
R2 = *Bathycoccus* sp.	0.0017	+		**40.0** ***	3.74^ns^	**11.97** *
CYANOBACTERIA	0.0014	+		**6.55** *	2.26^ns^	2.81^ns^
R4 = *Synechococcus*—type	0.0008			5.35^ns^	4.11^ns^	4.26^ns^
R7 = *Synechocystis*—type	0.0004			3.49^ns^	0.00^ns^	0.13^ns^
R3 = *Synechococcus*—type	0.0002	+		**10.74** *	4.34^ns^	**6.10** *
UNKNOWN						
R8	0.0007	-		**9.65** *	1.52^ns^	0.05^ns^

2-factor ANOVA; independent variables temperature and CO_2_, dependent variable asin√pi; species sorted within higher taxa according to the grand mean (without mesocosm 9) of biomass; R1, … R8 denote taxa whose abundance and biomass data were obtained by flow cytometry. Besides F-values also the sign of significant temperature and CO_2_ effects are shown; d.f. in all cases 1,7; Fp-int: F-ratio for interaction term; significance levels

^ns^:p>0.05

*: p<0.05

**: p<0.01

***: p<0.001.

^1^Most probably, in the flow cytometric analysis there were two, albeit overlapping clusters of *Chrysochromulina*, both were uncultured according to the genetic analysis and had overlapping sizes according to microscopy analysis.

**Fig 4:**
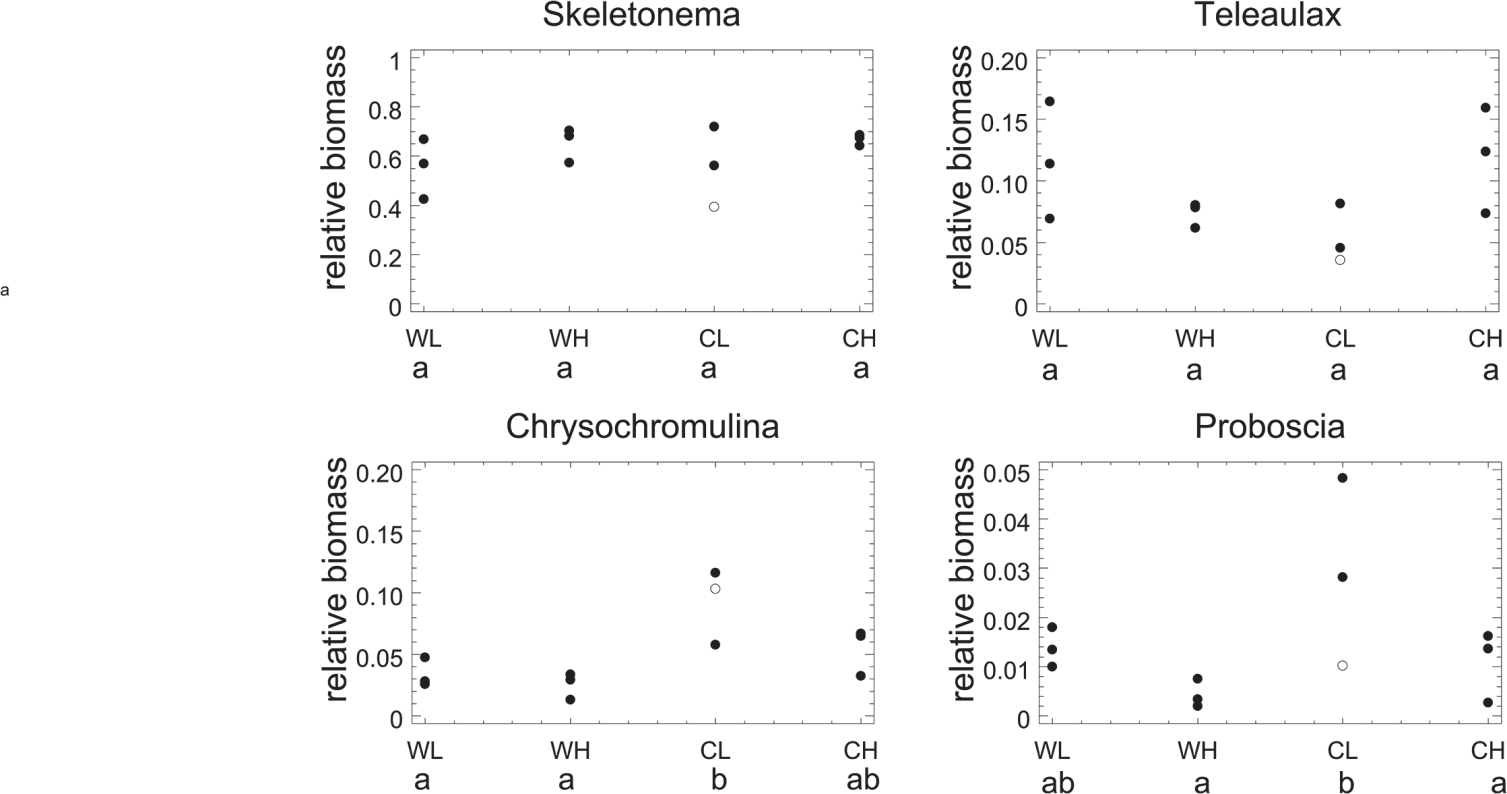
Relative biomass of selected species. Relative biomass (mean over entire duration of experiment) of the two most abundant species (*Skeletonema marinoi*, *Teleaulax acuta*) and of the most abundant species responsive to temperature (*Chrysochromulina* sp.) and the most abundant species responsive to CO_2_-enrichment (*Proboscia alata*). Treatment codes: WL: warm, low CO_2_, WH: warm, high CO_2_, CL: cold, low CO_2_, CH: cold, high CO_2_; codes for homogenous groups according to Tukey’s HSD: a, b.

The relative biomass (Table 1, Fig 4) of 8 species did neither respond to temperature nor to CO_2_. Among them were the 4 most important taxa in terms of biomass, the small-celled diatom *Skeletonema marinoi*, the cryptophyte *Teleaulax acuta*, and the large celled-diatoms *Coscinodiscus wailesii* and *Rhizosolenia setigera*. Together, they made up for 83.5% of total biomass (grand mean). Six species responded negatively to temperature, among them *Chrysochromulina* spp., the 5^th^ taxon in the ranking of biomass. 10 species responded positively to temperature. A significant negative response to CO_2_ was found in 7 species, while no significant positive response was found. Together, the species responsive to CO_2_ formed on average 4% of biomass.

### Cell size

**Fig 5:**
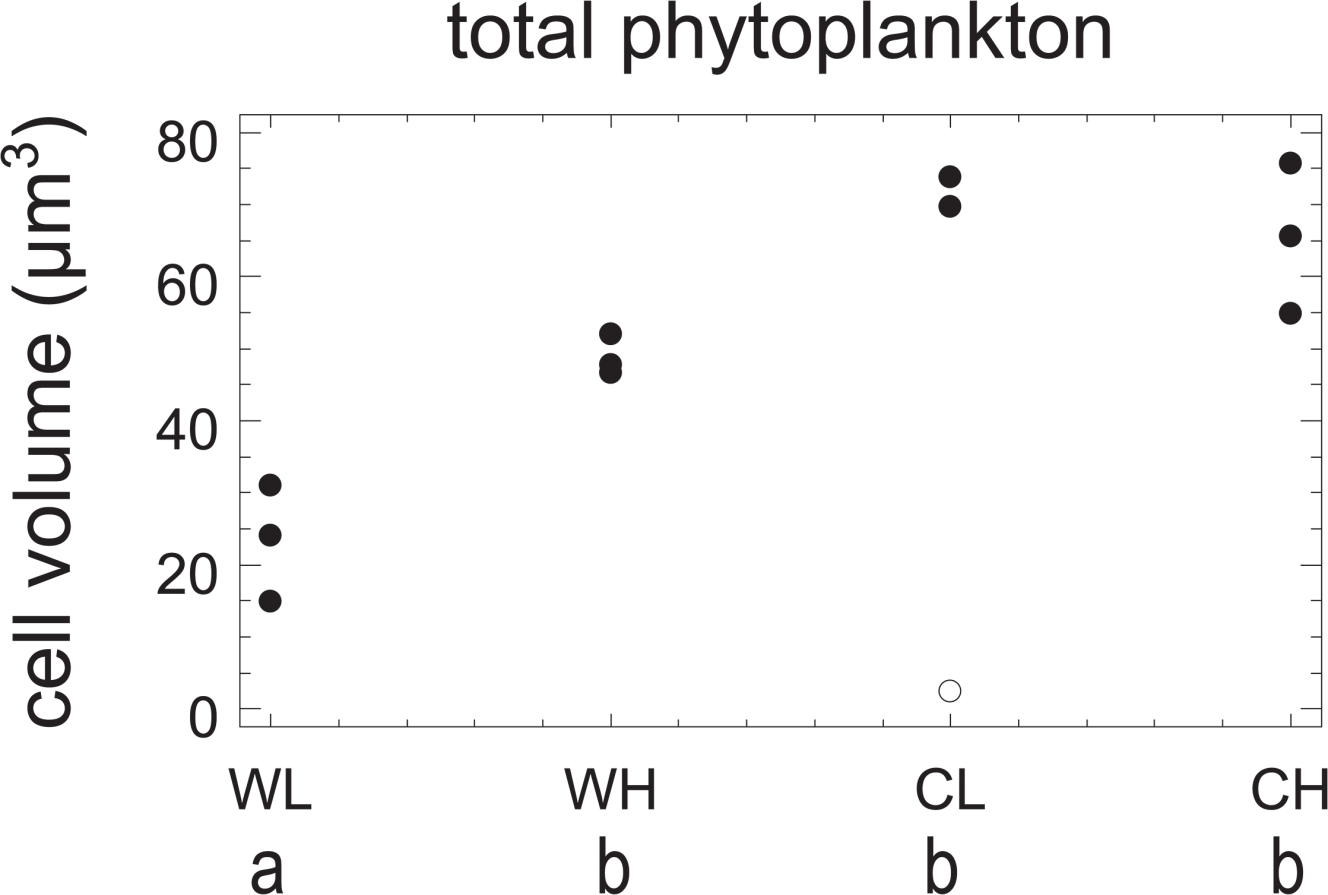
Community mean cell size. Response of community mean cell size (calculated from mean total biovolumes divided by mean cell numbers for each mesocosm) to the experimental treatments, mesocosm 9 shown by an open symbol. Treatment codes: WL: warm, low CO_2_, WH: warm, high CO_2_, CL: cold, low CO_2_, CH: cold, high CO_2_; codes for homogenous groups according to Tukey’s HSD: a, b.

**Fig 6:**
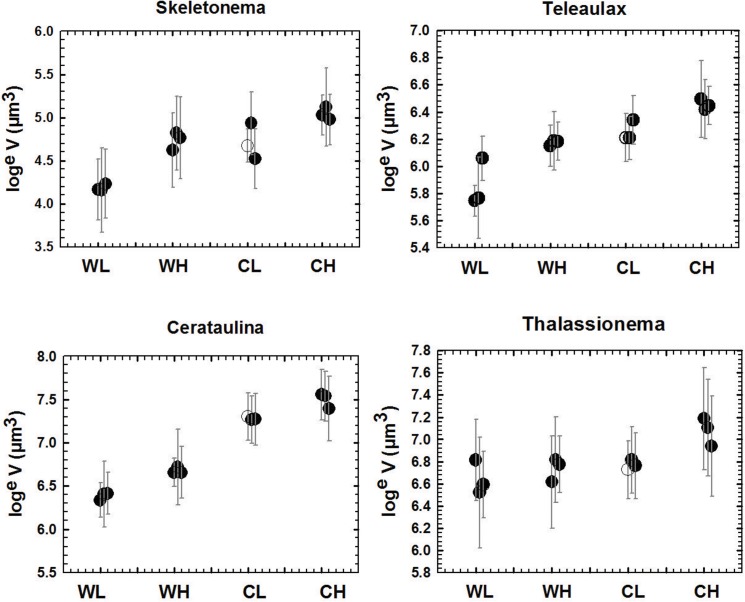
Cells size of selected species. Size response of 4 selected phytoplankton species to the experimental treatments, means and standard deviations (vertical lines) based on measurements of 20 cells per mesocosms. Mesocosm 9 shown by an open symbol. Treatment codes: WL: warm, low CO_2_, WH: warm, high CO_2_, CL: cold, low CO_2_, CH: cold, high CO_2_.

The mean cell size of total phytoplankton decreased significantly with warming and increased with CO_2_-enrichment (Fig 5, Table 2). Also the interaction term was significant. Fourteen phytoplankton species and 3 heterotrophic flagellates (the pico-sized *Bolidomonas*- like, and the nano-sized *Telonema subtilis* Griessmann and *Cryothecomonas* cf. *longipes* Schnepf & Kühn) were abundant enough for reliable size measurements (Fig 6, Table 2). Eleven phytoplankton species showed a significant, negative response to warming. There was no case of a significant positive response. Responses to CO_2_ were significantly positive for 8 phytoplankton taxa, significantly negative for the small phytoflagellate *Plagioselmis prolonga* Butcher and non-significant for 5 taxa. The two larger heterotrophic flagellate taxa showed a significant negative response to temperature and none responded to CO_2_ while the smallest heterotrophic flagellate showed no significant response to the experimental treatments.

**Table 2:** Cell size.

	V_mean_μm^3^	ES-temp	ES-CO_2_	F-temp	F-temp	F-int
Total phytoplankton						
logBV_mean_		-0.725	0.339	**29.2** ***	**6.38** *	**10.8** *
indidual taxa, algae (V; log μm^3^ cell^-1^)						
*Skeletonema marinoi*	121	-0.392	0.444	**20.8** **	**26.8** **	2.32^ns^
*Cerataulina pelagica*	1191	-0.851	0.276	**442.4** ***	**46.9** ***	0.42^ns^
*Thalassiosira nordenskioeldii*.	1402	-0.451	0.382	**45.9** ***	**32.7** ***	0.47^ns^
*Dactyliosolen fragilissimus*	4865	-0.715	0.409	**237.2** ***	**77.5** ***	0.24^ns^
*Rhizosolenia setigera*	160175	-0.403	0.398	**17.2** **	**13.0** **	0.91^ns^
*Guinardia flaccida*	5225	-0.140	0.188	**7.86** *	**13.0** **	0.34^ns^
*Thalassionema nitzschioides*	991	-0.294	0.238	**25.1** **	**19.6** **	3.26^ns^
*Teleaulax acuta*	507	-0.333	0.256	**31.4** ***	**16.0** **	0.94^ns^
R6 = *Chrysochromulina* spp.^1^	4.51	-0.751	-0.17	**54.5** ***	3.09^ns^	1.07^ns^
R5 = *Plagioselmis prolonga*	20.63	-0.049	-0.323	0.31^ns^	**7.38** *	2.49^ns^
R1 = *Ostreococcus* sp.	0.373	-0.020	0.003	**6.79** *	0.15^ns^	0.05^ns^
R2 = *Bathycoccus* sp.	0.456	-0.004	-0.001	0.01^ns^	0.00^ns^	0.21^ns^
R3 = S*ynechococcus*-type	0.357	-0.016	-0.004	2.73^ns^	0.18^ns^	0.00^ns^
R4 = S*ynechococcus*-type	0.356	-0.045	-0.019	**27.7** **	5.16^ns^	1.15^ns^
indidual taxa, heterotrophic flagellates (V; log μm^3^ cell^-1^)						
*Bolidomonas*—like	2.074	-0.021	0.031	0.84^ns^	1.96^ns^	0.58^ns^
*Telonema subtilis*	60.65	-0.624	0.0013	**200.6** ***	0.10^ns^	0.72^ns^
*Cryothecomonas* cf. *longipes*	182.4	-0.729	-0.027	**57.3** ***	0.08^ns^	0.012^ns^

2-factor ANOVA; independent variables temperature and CO_2_, dependent variable log cell volume (μm3); species sorted within higher taxa according to the grand mean (without mesocosm 9) of biomass; besides F-values also effect sizes (ES) are shown; d.f. in all cases 1,7; significance levels

^ns^: p>0.05

*: p<0.05

**: p<0.01

***: p<0.001.

^1^Because of the appearance of a larger, but overlapping *Chrysochromulina*-cluster towards the end of the experiment *Chrysochromolina*-sizes were taken already from day 12.

An analysis of the effect sizes of temperature and CO_2_ on the cell volumes of phytoplankton showed that E_T_ and E_C_ on size are themselves size dependent (expressed by the grand mean of cell volume, V_GM_):

$$E_{T}=-0.156-0.084log^{10}V_{GM};r^{2}=0.29;p=0.048;n=14$$

$$E_{C}=-0.033-0.092log^{10}V_{GM};r^{2}=0.54;p=0.0032;n=14$$

This means, that both the negative size effect of temperature and the positive size effect of CO_2_ are more pronounced for larger species. The larger heterotrophic flagellates also showed a negative size effect of temperature, but no size effect of CO_2_. The E_T_-cell size relationship for heterotrophic flagellates was marginally insignificant because of the low number of taxa included:

$$E_{T}=-0.088-0.375log^{10}V_{GM};r^{2}=0.99;p=0.064;n=3$$

## Discussion

### Phytoplankton biomass

The phytoplankton succession during the experiment took the typical course of transient autumn blooms in Kiel Bight, when after extended vertical mixing in Kiel Bight surface concentrations of nitrate, phosphate and silicate are saturating for algal growth and calm and sunny conditions permit a growth pulse of phytoplankton. Hypothesis 1 (lower biomass under warmer conditions) was confirmed while hypothesis 2 (higher biomass at higher CO_2_) was only partially confirmed, in our case for the warm mesocosms. The temperature effect agrees with a suite of spring experiments performed in the same mesocosm system [22,27,33] and a similar experiment in coastal waters of North Carolina [34,35]. However, one summer experiment using the Kiel mesocosm produced the opposite result [36]. The usual explanation for the temperature effect is a stronger increase of top-down factors (zooplankton grazing) with warming than of algal growth, because of the temperature-insensitivity of light limited photosynthesis [27,34,35]. The CO_2_-effect is consistent with the assumption that CO_2_ might be occasionally limiting for phytoplankton growth, while it is inconsistent with the assumption that CO_2_ is a stressor for the local phytoplankton community.

### Taxonomic shifts

Hypotheses 3 and 4 predicting taxonomic shifts in response to warming and CO_2_ addition were supported only to a small extent. Neither warming nor CO_2_ addition changed the relative biomass of the two major higher taxa (diatoms, cryptophytes) and of the 4 most important species. Shifts were only found for subdominant or rare species. The weakness of temperature effects was a surprise for us, because a 6°C temperature difference had strong effects on species composition in the spring bloom experiments in the same experimental systems in 5 successive years [22]. However, a shift from 9 to 15°C might have a smaller selection effect between species than a shift from 2 to 8°C. Interestingly, two summer experiments in the same facility [36,37] also revealed weak temperature effects on taxonomic composition. It is also possible that the different responses of subdominant/rare species could have been the precursor of dominance shifts if phytoplankton succession could have proceeded further.

The weakness of a CO_2_ effect on the dominant species agrees with an experiment at the Azores where differences in the relative biomass of dominant between the acidified and non-acidified treatments was usually <10% of total biomass. [17]. Contrary to a previous study [20] found a slight negative effect of CO_2_ on *Chrysochromulina* sp., this effect was not found for mean values in our experiment, but the decline of *Chrsochromulina* spp. during the final sampling intervals in the low CO2 treatments might hint in the same direction. Overall weak effects indicate similar adaptations to CO_2_ among the species coming from an environment with high natural CO_2_-fluctuations [21]. We cannot exclude that a stronger CO_2_ manipulation could have led to stronger effects, but it would have been beyond the atmospheric CO_2_-enrichment predicted for the end of the 21^st^ century even in the pessimistic IPCC-scenarios.

### Cell size

Hypothesis 5 predicted reduced cell sizes of phytoplankton and heterotrophic nanoflagellates under warmer condition while hypothesis 6 predicted an increase of cell sizes under CO_2_-enrichment only for phytoplankton. The general trend shrinkage of cell size with warming is in agreement with the majority of recent literature, which have analyzed cell size shifts within species [23], at the community level [38,39] or at both levels simultaneously [24,25]. However, the relative importance of shifts between and shifts within species differs from the previous papers, where shifts between species were by far the dominant factor. This can be shown if actual cell sizes in the individual mesocosms are replaced by the grand mean of each species, calculated community mean cell size still declines, but the effect size shrinks from -0.728 to -0.34, i.e. to less than the half. This is due to exceptional insensitivity of taxonomic composition to temperature in our experiment, while most of the previous studies have reported strong taxonomic effects of warming [22]. The CO_2_-effect on cell size bears some resemblance to the nitrogen effect in a recent study [25] where cell sizes strongly declined with the intensity of N-limitation. This is in agreement with the assumption of CO_2_-limitation and the well known effect that smaller cells suffer a smaller disadvantage, if diffusion of a limiting resource is the rate limiting effect (for CO_2_: [11]). However, we cannot exclude the alternative explanation that a better supply of CO_2_ led to an increased synthesis of non-protein components of biomass and thereby lead to an inflation of cell sizes. An increase share of non-protein biomass components would agree with previous findings of increased C: N consumption ratios under elevated CO_2_ [12]. The explanation by CO_2_-limitation would be supported if the positive size effect of CO_2_ would be absent under depletion by mineral nutrients precluding CO_2_-limitation. This was indeed the case in a small-scale (9 L) bottle experiment in the Okhostk Sea [40] where CO2-addition lead to a shift towards pico-cyanobacteria and picoplankton eukaryotes.

### Implications for the ocean acidification problem

We admit, that the global applicability of our findings is limited by the fact, that the Western Baltic Sea is an environment of high CO_2_ variability [21] and, therefore, phytoplankton already now have been selected for wide CO_2_ tolerance limits. However, wide fluctuations of CO_2_ are normal for productive ocean regions where the actual pCO_2_ in a water body is more strongly influenced by the balance between photosynthesis and respiration than by changes in the atmospheric pCO_2_ [19]. Because of the outstanding importance of the productive zones of the oceans for global primary production, we suggest that at least outside the zones of coccolithophore dominance phytoplankton is not the most sensitive component of the global ocean’s pelagic ecosystems. On the contrary, CO_2_ enrichment might have a mildly eutrophicating function at restricted regional and seasonal scales, such as in our experiment. However, we expect that the increased strength of thermal stratification, the spatial extension of the subtropical gyres and the resulting decreased supply of nutrients to the surface ocean in a warming world [41,42,43] will exceed any eutrophication effect of CO_2_, thus making climate warming the much stronger impact on the global pelagic system than ocean acidification.

In order to evaluate potential changes of the pelagic ecosystem in response to warming and ocean acidification it is also necessary to ask how phytoplankton effects will be transmitted to higher trophic levels, in particular to mesozooplankton which forms the direct link between primary production and fish production. A recent laboratory study with one phytoplankton species (the diatom *Thalsassiosira pseudonana*) and one mesozooplankton species (the copepod *Acartia tonnsa*) gave reason for concern, because it indicated an amplification of effects [44]. Neither of the two species suffered from a fitness loss if cultivated at elevated CO_2_, but *Acartia* showed a lowered egg production rate when fed with *Thalssiosira* cultured at elevated CO_2_ because of a reduced content of polyunsaturated fatty acids (PUFAs) in the food. In a follow-up study with a natural protists of phytoplankton and heterotrophic /protozoan assemblage as food base this amplification of effects could not be repeated [45]. Similarly, in this mesocosms experiment there was no indication of an adverse effect of CO_2_ on copepod feeding conditions [46]. Basically, the abundance of copepodids and adults at the end of the experiment showed the same pattern as phytoplankton biovolume: highest in the cold—high CO_2_ treatments (30–35 ind l^-1^), intermediate in the cold—low CO_2_ and warm—high CO_2_ (20–25 ind l^-1^) and lowest in the warm—low CO_2_ treatments (15–20 ind L^-1^). Also an analysis of fatty acids showed no indication of a nutritional inadequacy of phytoplankton under high CO_2_ [46].Thus, it seems that increasing complexity of the experimental community dampens adverse effects, as anticipated in the insurance hypothesis about biodiversity—ecosystem effects [47]. However, it will take more experiments with different plankton communities to decide whether food web interactions act as “amplifiers” or “shock absorbers” of external forcing.
